# Development and evaluation of a point-of-care ultrasound curriculum for paramedics in Germany – a prospective observational study and comparison

**DOI:** 10.1186/s12909-024-05816-1

**Published:** 2024-07-29

**Authors:** Christopher Jonck, Andreas Michael Weimer, Beatrice Fundel, Wolfgang Heinz, Daniel Merkel, Hendrik Fiedel, Carlotta Ille, Roman Kloeckner, Holger Buggenhagen, Tim Piepho, Johannes Weimer

**Affiliations:** 1 Rudolf Frey Learning Clinic, University Medical Center of the Johannes Gutenberg University Mainz, Langenbeckstraße 1, Mainz, 55131 Germany; 2 Center of Orthopedics, Trauma Surgery, and Spinal Cord Injury, University Hospital Heidelberg, Heidelberg, Germany; 3 Department for Emergency Medicine, Hospital Maria Hilf Krefeld, Krefeld, Germany; 4 Department for Internal Medicine, Helios Klinik Rottweil, Rottweil, Germany; 5 Brandenburg Medical School Theodor Fontane (MHB), BIKUS - Brandenburg Institute for Clinical Ultrasound, Neuruppin, Germany; 6 German Red Cross, DRK Rettungsdienst in Der Region Hannover gGmbH, Hanover, Germany; 7 Institute of Interventional Radiology, University Hospital Schleswig-Holstein - Campus Lübeck, Lübeck, Germany; 8 Department of Anaesthesiology and Intensive Care, Brothers of Mercy Hospital, Trier, Germany; 9 Department of Internal Medicine I, University Medical Center of the Johannes Gutenberg University Mainz, Mainz, Germany

**Keywords:** Point-of-care sonography, POCUS, Ultrasound training, Curriculum development, Imaging, Sonography, Ultrasound training, Blended learning, Paramedic, Emergency medical service, Emergency medical technician, Emergency medicine, Prehospital

## Abstract

**Background:**

Point-of-care ultrasound (POCUS) is steadily growing in use in prehospital emergency medicine. While currently used primarily by emergency physicians, POCUS could also be employed by paramedics to support diagnosis and decision-making. Yet to date, no paramedicine-targeted POCUS curricula exist in Germany. Furthermore, given time and resource constraints in paramedic training, it is unclear whether paramedics could feasibly learn POCUS for prehospital deployment. Hence, this study outlines the development and implementation of a comprehensive POCUS curriculum for paramedics. Through this curriculum, we investigate whether paramedics can attain proficiency in POCUS comparable to other user groups.

**Methods:**

In this prospective observational study, we first developed a blended learning-based POCUS curriculum specifically for paramedics, focusing on basic principles, the RUSH-Protocol and ultrasound guided procedures. Participants underwent digital tests to measure their theoretical competence before (T1) and after the digital preparation phase (T2), as well as at the end of the on-site phase (T3). At time point T3, we additionally measured practical competence using healthy subjects and simulators. We compared the theoretical competence and the practical competence on a simulator with those of physicians and medical students who had also completed ultrasound training. Furthermore, we carried out self-assessment evaluations, as well as evaluations of motivation and curriculum satisfaction.

**Results:**

The paramedic study group comprised *n* = 72 participants. In the theoretical test, the group showed significant improvement between T1 and T2 (*p* < 0.001) and between T2 and T3 (*p* < 0.001). In the practical test on healthy subjects at T3, the group achieved high results (87.0% ± 5.6). In the practical test on a simulator at T3, paramedics (83.8% ± 6.6) achieved a lower result than physicians (*p* < 0.001), but a comparable result to medical students (*p* = 0.18). The results of the study group’s theoretical tests (82.9% ± 9.2) at time point T3 were comparable to that of physicians (*p* = 0.18) and better than that of medical students (*p* < 0.01). The motivation and attitude of paramedics towards the prehospital use of POCUS as well as their self-assessment significantly improved from T1 to T3 (*p* < 0.001). The overall assessment of the curriculum was positive (92.1 ± 8.5).

**Conclusion:**

With our tailored curriculum, German paramedics were able to develop skills in POCUS comparable to those of other POCUS learners. Integration of POCUS into paramedics’ training curricula offers opportunities and should be further studied.

**Supplementary Information:**

The online version contains supplementary material available at 10.1186/s12909-024-05816-1.

## Introduction

### Background

Emergency ultrasound has become integral to the diagnosis of critically ill patients [1]. In emergency situations, important clinical questions can be answered quickly based on ultrasound findings to enable targeted follow-up diagnostics and treatments [2]. Ultrasound performed and interpreted directly at the bedside is known as point-of-care ultrasound (POCUS) [3].

With technical advances in handheld ultrasound devices, POCUS is now increasingly used in prehospital emergency medicine, where it may improve patient care, especially for trauma patients [4–7]. In Germany’s physician-based emergency medical service (EMS) system, emergency physicians are making increasing use of POCUS [8–11]. POCUS training for this user group usually takes place during residency through participation in certified courses. The training curricula of these courses are based on recommendations of the relevant professional associations [8, 12]. Meanwhile, medical schools are increasingly also teaching a fundamental understanding of sonographic anatomy and POCUS skills based on national and international recommendations [13–18]. These developments have made POCUS much more accessible for emergency physicians.

By contrast, the use of POCUS by paramedics is less well-established and is the subject of current research [19–23]. Despite the heterogeneous definition of the term paramedic, existing studies have shown that the use of POCUS by paramedics is feasible for the most part, although the influence on patient outcomes remains uncertain [24, 25]. In Germany, paramedics lack the authorisation to perform POCUS either independently or in cooperation with emergency physicians due to a lack of equipment, training curricula, and for medico-legal reasons. However, POCUS training for paramedics would enable them to support emergency physician colleagues in critical situations. Equally, paramedics trained to use POCUS unaided could optimize decision-making in patient care without physicians [21, 22, 25, 26]. Given the ongoing debate about academization of the existing three-year paramedic training programme in Germany and the expansion of skillsets to reduce reliance on prehospital emergency physicians, training paramedics in POCUS seems extremely promising. It offers a significant possible enhancement of diagnostic procedures, healthcare system efficiency, and patient care [27].

### Research question and hypothesis

Several studies have dealt with the use of POCUS by paramedics, yet only a handful have focussed on the didactic curricula required for effective implementation [23, 28–30]. Generally, these didactic studies centre on specific forms of sonography (e.g. e-FAST or thoracic sonography) and vary considerably in course content, scope of learning, duration of training, and didactic approach [31]. The courses developed were often not explicitly directed at the training of paramedics nor accounted for limited prior knowledge of ultrasound-related basic principles such as anatomy and physiology; instead, they mainly targeted physicians [32]. This study is therefore concerned with the development and evaluation of a target group-specific curriculum for paramedics, covering anatomy and ultrasound basics as well as practical training of different applications (e.g. eFAST, RUSH, ultrasound guided procedures). It asks whether theoretical and practical skills in POCUS can be developed through this curriculum by comparing measurable learning outcomes with those from other user groups. A further aim of the study is to ascertain curriculum satisfaction and explore the future prospects of POCUS teaching in paramedicine. The study’s primary hypothesis posits that the participants, after working through the target group-specific curriculum, will improve their theoretical and practical skills in POCUS and achieve a level of competence on par with other user groups. Additionally, we anticipate that paramedics will embrace the curriculum, exhibit high motivation and a positive attitude, and envision future prospects for integrating POCUS into their professional practice.

## Materials and methods

### Study procedure and endpoints

**Fig. 1:**
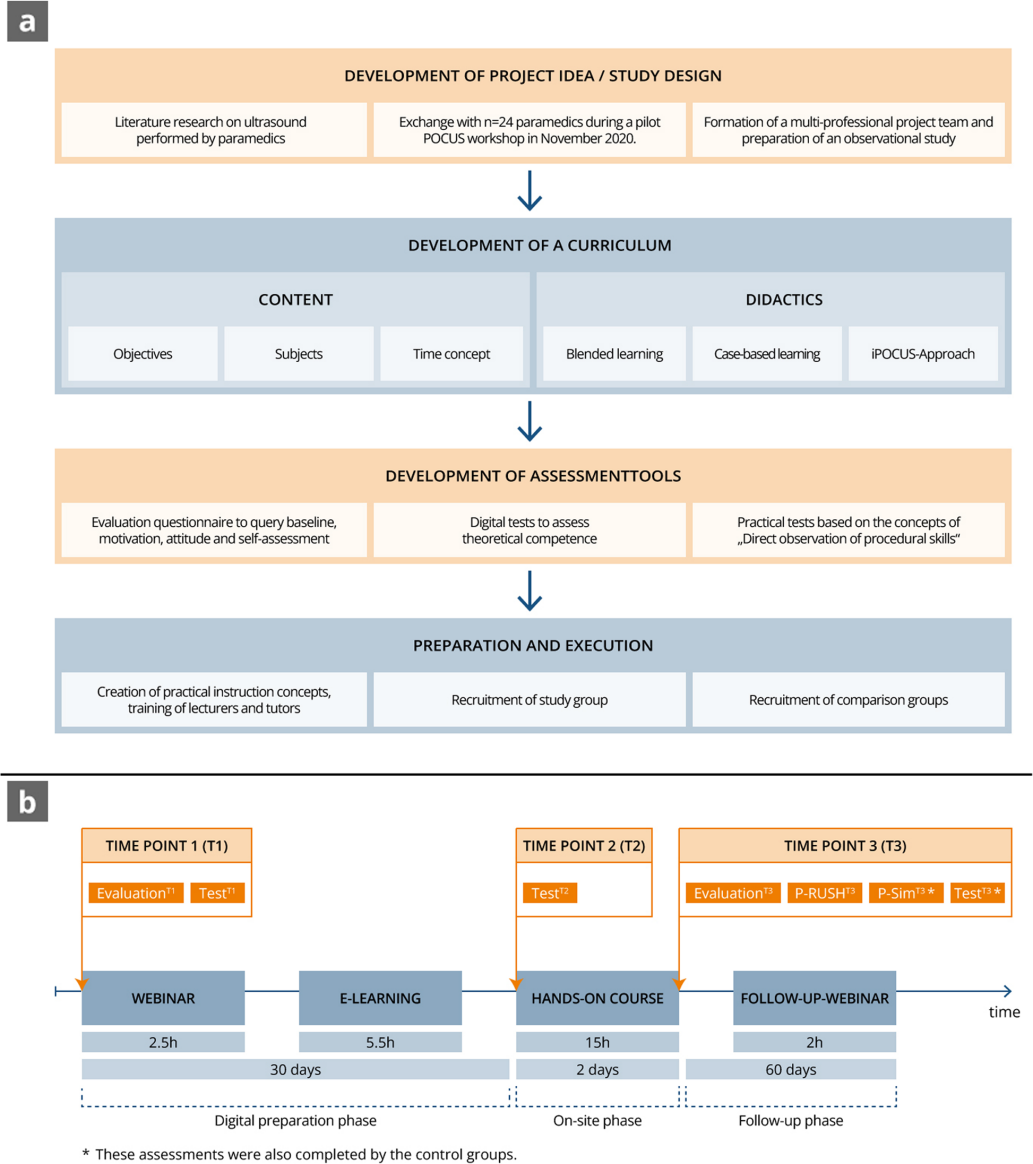
Study development and implementation process. **a** Overview of the process of study planning and curriculum development.; **b** Breakdown of the timings of the paramedic-specific curriculum and the time points of the measurements

This prospective observational study took place in Mainz, Germany, from January 2022 to December 2022. After developing a comprehensive POCUS curriculum for paramedics, we measured theoretical and practical skill acquisition and acceptance of the curriculum (Fig. 1). To do this, at three time points (T1: before the start of the preparation phase, T2: at the end of the preparation phase, T3: at the end of the on-site phase) we used theoretical tests (Test^T1^, Test^T2^, Test^T3^), practical tests (P-Sim^T3^, P-RUSH^T3^), and evaluations (Evaluation^T1^, Evaluation^T3^) [33]. With the aid of two control groups consisting of physicians and medical students, we compared theoretical and practical skills at time point T3 and analysed possible influencing factors. The primary study outcome is the significant gain in theoretical competence of paramedics and the comparability of their competence with the control groups. Secondary outcomes are the significant subjective improvement in competence, acceptance of the curriculum, high motivation, and positive attitude towards the preclinical use and future implementation of POCUS.

### Recruitment and inclusion criteria

Through online advertisements, we invited paramedics throughout Germany to participate in the curriculum and the study. The inclusion criteria for the study group were completion of paramedic training, fully completing the curriculum and its assessment tools at the appropriate time points, and consent to participate in the study. The defined inclusion criteria for the control groups were full participation in the training, full completion of the assessment tools, and consent to participate in the study.

### Curriculum

#### Curriculum development and implementation

Based on established practices in medical curriculum development, we first created a target group-specific curriculum (Fig. 1) to address the individual needs of paramedics and to deduce suitable teaching strategies [34]. The learning objectives and content (Table 1) were defined by emergency physicians, paramedics, educators, and ultrasound experts based on the paramedic training curriculum, the DEGUM [German Society of Ultrasound in Medicine and Biology] curriculum for basic training in emergency sonography, and previously published studies [28, 32, 35, 36]. The curriculum comprised a total of 25 h and was divided into a digital preparation phase (live webinar and e-learning), an on-site phase (theory units and practice units), and a digital follow-up phase (live webinar and e-learning). Key didactic elements were the use of blended learning [37], the integration of case-based learning using five case scenarios (Supplement 1), and the development and use of a structured approach to an ultrasound examination with the help of the mnemonic “iPOCUS” [38]. The individual letters stand for Indication (I), Positioning (P), Orientation (O), Correction (C), Ultrasound examination (U) and Save & Speak (S).

**Table 1:** Phases, implementation and content of the curriculum. *Modified RUSH Protocol: Exclusion of the echocardiographic sections

Phase	Implementation	Topic	Content
**Preparation**	Live-Webinar (Lecture)	Anatomy	Vascular anatomy (inferior vena cava, abdominal aorta) Body cavities (pericardial, pleural and peritoneal cavities) Thorax (chest wall, lungs, heart) Topography of the abdominal organs
	E-Learning (short videos and quizzes)	Basics	Principles of sonography Sound wave theory Impedances, image generation and modes Axis understanding (sagittal + transversal) Artifacts and their origin Transducer types and image optimization Organ morphology Terminology and documentation
		Examinations	Basics of organ examination Standard sections Interpretation of normal findings Measurements and standard values
**On-site**	Hands-on course (lectures and practical exercises)	Examinations	Examination of Inferior vena cava Examination of the Abdominal aorta Examination of the body cavities Examination of the kidney Examination of the lungs Examination of the heart (subxiphoid view) Examination of the deep leg veins (3 points)
		Protocols	Extended Focused Assessment with Sonography in Trauma (eFAST) Modified Rapid Ultrasound for Shock and Hypotension (RUSH)*
		Procedures	Ultrasound-guided peripheral intravenous access
		Pathologies	Heart failure Aortic aneurysm, aortic rupture, aortic dissection Free fluid Urinary retention Interstitial syndrome, pneumonia, pneumothorax, pleural effusion Pericardial effusion, pericardial tamponade, right heart strain Deep venous thrombosis (DVT)
**Follow-Up**	Live-Webinar (Lecture)	Repetition	Anatomy Pathologies

In the preparation phase, the participants attended a webinar, then worked through 70 short videos and 42 single-choice questions in a specially designed e-learning program. The on-site phase involved a brief repetition of basics, discussion of pathologies, and practice of the examinations at workstations (Supplement 2a). One tutor for each four participants gave instruction at the workstations using a pre-defined checklist. Each participant had at least 84 min of personal practice time on the ultrasound machine (+ 30 min of practical assessments) during the entire on-site phase (Supplement 2b). Different stationary and handheld ultrasound devices as well as an ultrasound simulator (Vimedix, CAE Healthcare, USA) were employed. For the course follow-up, participants could access the e-learning program and had the option of attending a follow-up webinar to repeat and consolidate their knowledge. The lecturers and tutors were physicians and paramedics who had practical and teaching experience in ultrasound diagnostics and training. They had received additional training in preparation for their educational activity.

### Assessment tools

#### Evaluations

Evaluation^T1^ and Evaluation^T3^ measured baseline characteristics, prior experience, motivation and attitude, personal learning objectives, and required self-assessment. The evaluations consisted of categorical (free text, drop-down menu) and continuous items. Evaluation^T3^ additionally assessed participants’ satisfaction with the curriculum.

#### Theoretical tests

Test^T1^ and Test^T2^ were conducted in order to measure the development of theoretical competence. Each involved 30 tasks for a maximum of 66 assessment points (AP) over 45 min. The tasks involved texts, images and video clips and allowed single-choice or free-text answers on smartphones or tablets. Participants were tested on anatomy (17 AP), basic ultrasound principles (17 AP), orientation (5 AP), and structure detection (27 AP). The final Test^T3^ additionally contained ten tasks (40 AP) on pathology detection with a total maximum time of 60 min and 106 achievable APs (Supplement 3).

#### Practical tests

The practical test forms were specially developed from the Direct Observation of Procedural Skills (DOPS) test [33, 39]. We used these forms to test three focused organ examinations on an ultrasound simulator (P-Sim^T3^, 66 AP). The assessment was based on the previously taught iPOCUS mnemonic. At least one correct indication (i), selection of the correct landmark for positioning (P), proper orientation with the transducer (O), adequate image correction with depth, gain, focus (C), thorough ultrasound examination (U) and saving of an image or clip (S) were measured. We additionally assessed the subjects’ use of a modified Rapid Ultrasound for Shock and Hypotension (RUSH) protocol on a healthy subject (P-RUSH^T3^, max. 315 AP, performance time 10 min; see Supplement 4) [40]. All practical tests were conducted by one examiner per participant, with a total of five practical examiners being involved in the study.

### Control groups

The physician control group comprised participants from two DEGUM-certified basic ultrasound courses. The student control group consisted of two semesters of third-year medical students completing a peer-to-peer ultrasound course [41]. The course curricula used for both groups were similar to the curriculum for paramedics in terms of structure, duration, and content. Participants of both control groups underwent a baseline survey on baseline characteristics and prior experience, then completed Test^T3^ or P-Sim^T3^ at the end of their courses (Supplement 5a).

### Data collection and statistical methods

Data collection was carried out using the survey tool LimeSurvey (LimeSurvey GmbH, Germany) and written questionnaires. All statistical analyses were performed in Rstudio (R 4.0.3). Binary and categorical baseline variables were given as absolute numbers and percentages. Continuous data were given as median and interquartile range (IQR) or as mean and standard deviation (SD). Continuous items (Likert scales 1–7) were mathematically (using the 'min–max scale') transformed to a scale from 0 to 1 and multiplied by 100 to obtain data in per cent. The max scale was used, which means that 1 is the lowest and 100 is the highest score. Categorical variables were compared using Fisher’s exact test and continuous variables using the T-test or the Mann–Whitney U test. Additionally, parametric (ANOVA) and non-parametric (Kruskall-Wallis) analyses of variance were calculated and further explored with pairwise post hoc tests (T-test or Mann–Whitney U). Prior to the inference statistics, we conducted pairwise correlations of variables and plotted the correlations’ effect sizes and significances. Multivariate linear regression models were constructed to compare the influence of individual factors on the results of the tests. *P*-values < 0.05 were considered statistically significant.

## Results

### Baseline characteristics

In total, our analysis included data from *n* = 337 participants. This figure comprised the paramedic study group (*n* = 72) and the two control groups consisting of physicians (*n* = 132) and students (*n* = 133). The in- and exclusion process is illustrated in Supplement 5b. The mean age in the study group was 31.6 ± 9.5 (physicians: 31.8 ± 5.5, students: 24.6 ± 3.9) and 18.1% were female (physicians: 59.1%, students: 67.7%). Among the study group, 20.8% had previously attended one or more ultrasound courses (physicians: 32.6%, students: 15.0%), and 37.5% (physicians: 99.2%, students: 29.6%) reported experience of independently performing ultrasound examinations. In the study group, 65 participants (90.3%) were qualified as paramedics and seven (9.7%) were emergency medical technicians (EMTs). Supplements 6 and 7 show the detailed characteristics of the study and control groups.

### Results of the subjective evaluation

The total evaluation score (Likert scale 1–7, transformed into percentages) for motivation and general attitude to the subject of POCUS in EMS was already high at the start of the training and showed further significant improvement during the training (Evaluation^T1^: 81.6 ± 11.5; Evaluation^T3^: 87.8 ± 11.4; *p* < 0.01) (Supplement 8). The future integration of POCUS into the training and work of paramedics garnered approval (Evaluation^T3^: mean 94.4). The self-assessment of personal skills was initially low but improved significantly (Evaluation^T1^: 29.7 ± 20.1; Evaluation^T3^: 70.9 ± 14.5; *p* < 0.01). This improvement was observed across the theoretical and practical self-assessments, and across all the sub-items queried. Furthermore, participants achieved their defined learning objectives as a result of the training (Evaluation^T3^: 82.0 ± 11.4). In the study group’s evaluation of the curriculum, all the items queried were rated highly (> 87.2%). Table 2 presents the assessment of the curriculum in detail.

**Table 2:** Results of Evaluation^T3 ^relating to queried items regarding satisfaction with the elements of the curriculum. Continuous items (likert scale transformed to a scale from 0 to 1 and multiplied by 100; 0 = not at all true, 100 = completely true) presented after transformation into percentages as mean and standard deviation (SD)

Item	Mean ± SD in %
Total score	91.6 ± 6.3
**Webinar (overall score)**	89.6 ± 12.6
General satisfaction with the webinar	91.3 ± 12.0
Content	92.3 ± 12.5
Increase in learning success through webinar	85.5 ± 20.2
Future relevance of webinar	93.9 ± 13.9
Increased motivation through webinar	84.9 ± 19.1
**E-Learning (overall score)**	93.9 ± 5.4
General satisfaction with the e-learning	93.3 ± 9.6
Content	95.3 ± 9.0
Methodology	95.9 ± 8.1
Video quality	96.6 ± 8.5
Audio quality	97.2 ± 8.5
Duration	92.3 ± 15.0
Quizzes	81.3 ± 21.0
Preparation benefits	93.3 ± 10.2
Personal knowledge gain	96.3 ± 8.7
Future relevance of e-learning	97.7 ± 6.7
Increased motivation through e-learning	93.7 ± 12.0
**Concept (overall score)**	92.1 ± 8.5
Blended learning	92.9 ± 14.2
General satisfaction with the concept	96.1 ± 8.4
Clarity of the concept	95.1 ± 9.1
Presentation of learning objectives	95.1 ± 9.7
Extent of content covered	92.1 ± 11.0
Achievement of learning objectives	90.0 ± 12.7
Use of case studies	90.1 ± 15.6
Teaching material	93.6 ± 10.8
Stringency of digital preparation for classroom course	93.5 ± 11.3
Adequate preparation for theoretical tests	84.7 ± 18.0
Adequate preparation for practical tests	89.7 ± 14.5
**On-site phase (overall score)**	90.1 ± 8.5
Theoretical content	90.3 ± 12.5
Practical content	95.5 ± 7.9
Ratio of theory to practice	93.3 ± 10.5
Sufficient time for practical training	77.6 ± 20.2
Sufficient time for theory repetition	93.9 ± 11.5
**iPOCUS Approach (overall score)**	91.5 ± 11.2
General satisfaction with the approach	94.1 ± 10.1
Suitable learning tool	89.3 ± 14.0
Applicability in preclinical use	89.9 ± 14.5
Future relevance of iPOCUS for learning ultrasound	92.7 ± 14.6
**Assessments (overall score)**	87.2 ± 16.3
General satisfaction with DOPS	89.4 ± 15.7
Increased competence through assessments	86.5 ± 18.7
Increased motivation through assessments	85.7 ± 25.1

### Results of the objective tests

#### Competence comparison

**Fig. 2:**
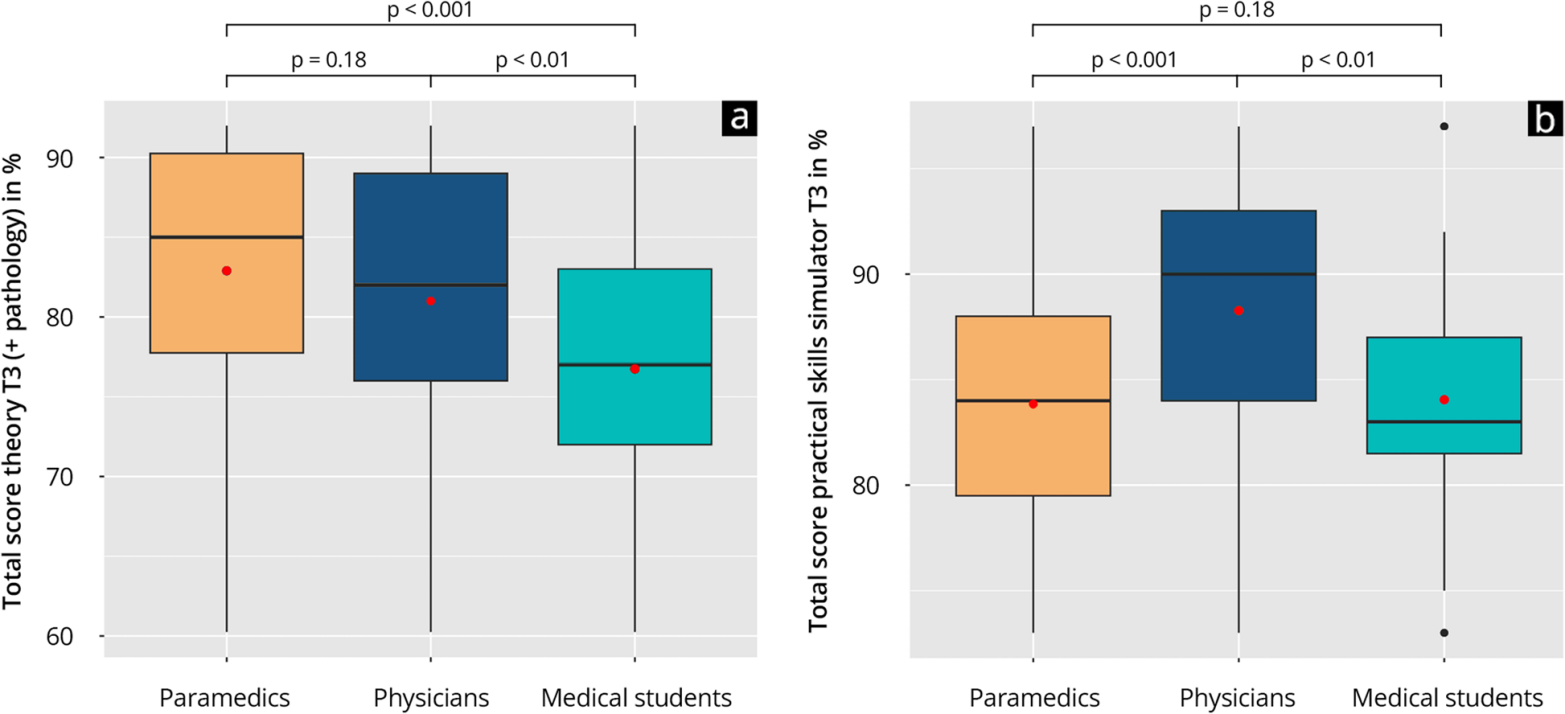
Competence comparison at time point T3. **a** Results of the theoretical test^T3^; **b** Results of the practical test P-Sim^T3^

Figure 2 and Supplement 9 show the detailed results of the competence comparison. In the total score for the theoretical Test^T3^, paramedics (82.9 ± 9.2) achieved a comparable result to physicians (81.0 ± 8.7; *p* = 0.55) without a significant difference. Paramedics achieved a significantly higher result than the group of students (76.7 ± 8.5; *p* < 0.001). In the practical test on the simulator (P-Sim^T3^) all groups achieved high percentage ranges (> 80%). Paramedics (83.8 ± 6.6) achieved a significantly lower result than physicians (88.3 ± 6.1, *p* < 0.001), but a non-significantly different result compared to medical students (84.1 ± 6.2, *p* = 0.86).

#### Development of theoretical competence by paramedics

**Fig. 3:**
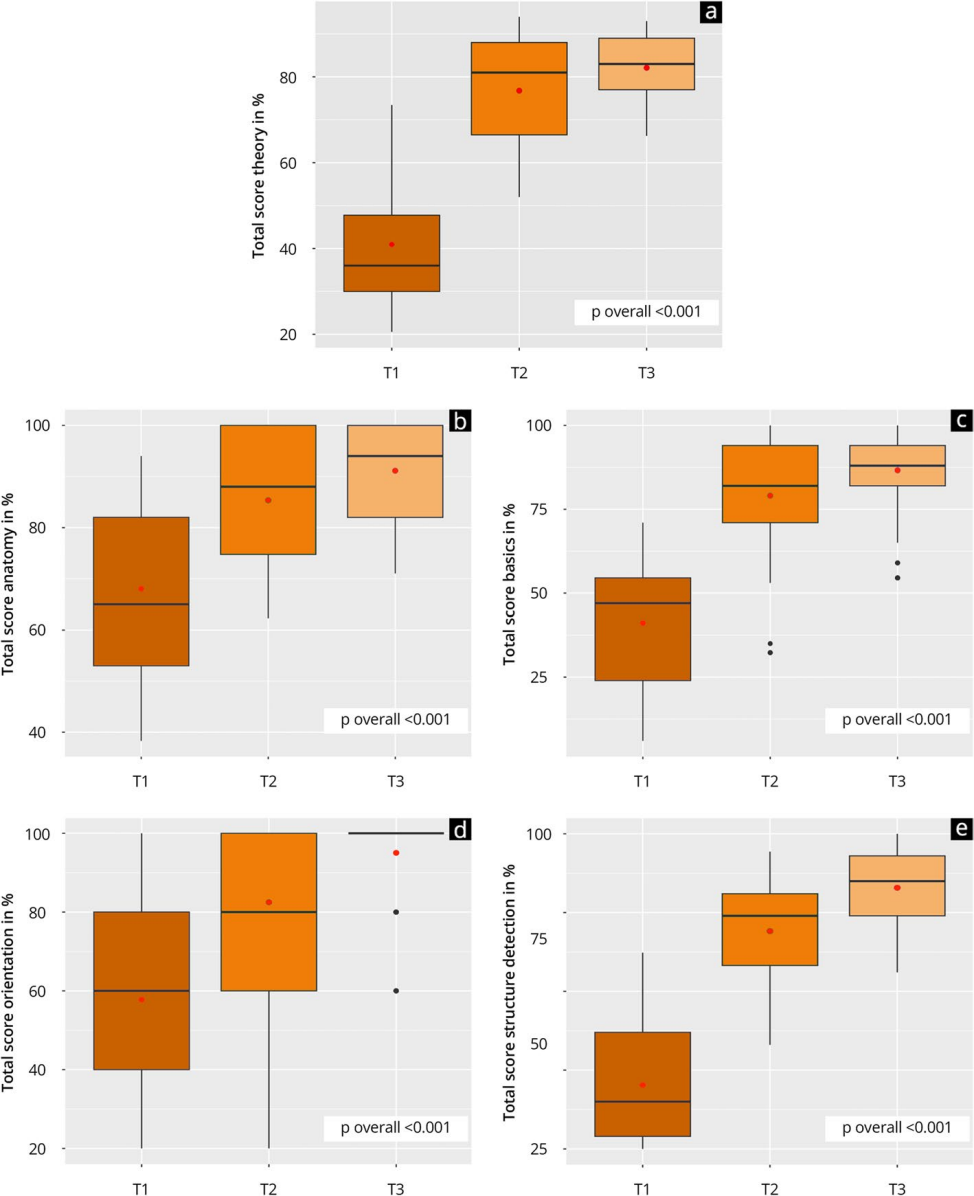
Results of the theoretical skills acquisition of paramedics over the study time points T1-T3. The boxplots visualize the results of the total scores (**a**) as well as the individual skill areas anatomy (**b**), basics (**c**), orientation (**d**), and structure detection (**c**); red dot = mean

Figure 3 shows the development of theoretical competence in the study group over various time points. The results improved significantly (*p* < 0.001) over the observation period from T1 (41.3 ± 16.0) over T2 (76.5 ± 14.5) to T3 (T3: 82.7 ± 9.7). This improvement was consistent across all the individual skill areas assessed in the tests. At time point T1, results were lower than 60% in all the skill areas except anatomy. At time point T2, paramedics achieved results of at least 68%. At time point T3, the group achieved results greater than 82% in all the individual skill areas. The most substantial increase throughout the observation period occurred in the skill area of structure detection (T1: 21.5 ± 23.0; T3: 82.9 ± 14.5; *p* < 0.001).

#### Practical competence of paramedics

**Fig. 4:**
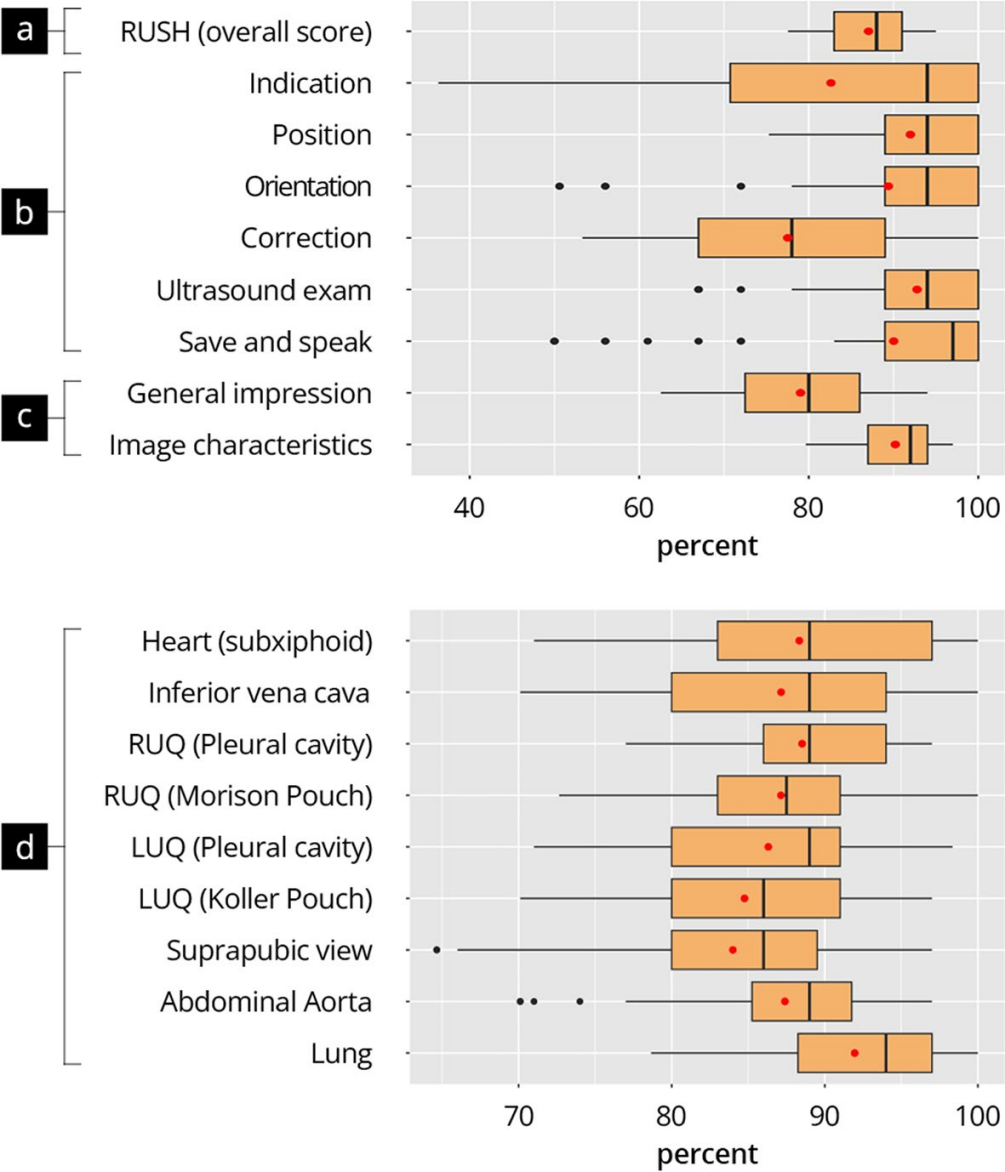
Results of the practical testing P-RUSH^T3^. The boxplots visualize the results of a simplified RUSH protocol on a healthy subject in terms of total score (**a**), individual examination steps by the iPOCUS approach (**b**), aspects additionally assessed by the examiner (**c**) and the individual views (**d**). RUQ = right upper quadrant, LUQ = left upper quadrant. Red dot = mean

In the practical testing of the RUSH protocol (P-RUSH^T3^), paramedics achieved total score results in high percentage ranges (87.0 ± 5.6). This strong performance extended to all the tested individual sonographic views and iPOCUS skills (> 83%). The best results were achieved in the views “Lung” (91.6 ± 7.0*)*, “Heart (subxiphoid)” (88.3 ± 8.9), and “Right Upper Quadrant (RUQ) pleural cavity” (88.4 ± 6.6). The results were lower for the tests of “Left upper quadrant (LUQ) Koller Pouch” (84.4 ± 9.6) and “Suprapubic view” (83.0 ± 12.5). Figure 4 shows the detailed results of the examinations.

#### Analysis of influencing factors (regression) and correlations

In a multivariate linear regression analysis of the performance of the study group for the total score of Test^T1^, “prior attendance of at least one ultrasound course” was identified as a significant influencing factor for a better test result (β = 11.2; *p* = 0.034). We observed no significant influencing factors for the results of Test^T2^, Test^T3^ and P-Sim^T3^. A high score in Test^T3^ was established as an influencing factor for a better result in P-RUSH^T3^ (β = 0.24; *p* = 0.002).

A regression model incorporating all groups and baseline characteristics tended to detect “belonging to the group of physicians” as an influencing factor for a high result in P-Sim^T3^ (β = 3.53; *p* = 0.068).

The “number of prior POCUS examinations done” was a significant influencing factor for a high result in Test^T3^ over all groups (β = 1.02; *p* = 0.046). Furthermore, “belonging to the group of students” was found to be an influencing factor for a significantly worse result in Test^T3^ (β = -7.34; *p* < 0.01).

In the correlation calculations, there were moderate to strong correlations among the objective theoretical and practical performances (*r* = 0.3–0.43; *p* < 0.001). However, the subjective and objective results only correlated strongly with each other at time point T1 (*r* = 0.43; *p* < 0.01).

## Discussion

This is the first prospective observational study focusing on the development and evaluation of a tailored point-of-care ultrasound curriculum for German paramedics that also compares the competence outcomes of paramedics with those of other user groups. After completing the curriculum, paramedics demonstrated proficiency in POCUS that was comparable to physicians and medical students who had also completed similar ultrasound training. Furthermore, paramedics participated in the curriculum with enthusiasm, displaying motivation and a positive attitude towards future POCUS training and application.

### Relevance of the study

The significance of this study stems from its departure from previous research focussed primarily on paramedics’ direct use of POCUS on patients [19, 21, 22, 25, 29, 42–49]. Other prior studies focused on POCUS education for paramedics have relied on established curricula aimed at physicians, overlooking paramedics’ unfamiliarity and inexperience with POCUS in prehospital settings [23, 28, 30–32]. Since it has not yet been conclusively shown that paramedics can learn POCUS, we assumed that the achievement of proficiency can only be developed on the basis of a specific curriculum. Therefore, our study developed a tailored curriculum for paramedics, carried out a comprehensive analysis of skill acquisition, and, for the first time, compared this with other POCUS learners. This approach furnished detailed information about the development of competence among paramedics in POCUS.

### Discussion of the competence comparison and competence development

Measuring the level of competence was an important endpoint in several studies published to date [23, 30]. Our study measured competence by conducting theoretical and practical tests. While other studies mainly observed and interpreted the absolute results of paramedics [23], our study managed to show that the final theoretical and practical skills were at a similar level to those of physician and medical student POCUS learners. Although the interpretation of the competence comparison is limited by the different curricula that the control groups completed, our competence comparison makes it possible to classify the results achieved by paramedics. Only one other study by Brook et al. has so far used a similar methodology, finding that paramedics achieved a higher competence level in the area of image interpretation than *n* = 2 ultrasound experts [50]. While the implications of Brook et al.’s results are difficult to classify, our comparative methodology, involving a significantly higher number of participants, also suggests that paramedics constitute a suitable target group for learning POCUS.

Previous studies have employed theoretical tests to demonstrate paramedics’ ability to develop theoretical skills after completing a curriculum [23, 30, 42, 50–57]. In our study, we also observed a significant development in theoretical competence, reaching a substantial level of final theoretical proficiency. While most studies conducted two measurements of theoretical competence, usually before and after completing a curriculum [30, 55, 56, 58], our study design, with a total of three assessment time points, enabled us to gain detailed information about competence development. Given the low test results observed before studying the curriculum, we can assume our study group had limited prior experience with POCUS [30]. Despite the internationally heterogeneous training and definition of “paramedics”, our initial results do coincide with those of other studies [30, 56, 57]. Furthermore, our study showed that paramedics could achieve a significant gain in theoretical competence through the digital preparation phase [30]. The final test results (approx. 83%) coincide with the results of other studies, although our study looks at a much larger population [23, 30, 56]. We can conclude that it is possible for paramedics to build up competence with a tailored POCUS curriculum, and a blended learning-based approach is appropriate in this teaching context.

In addition to theoretical tests, we also conducted practical tests in which paramedics achieved high results (approx. 87%). This finding is in line with the results of previous studies using practical testing formats [23, 29, 30, 50, 51, 54, 56, 59, 60]. While most studies used ultrasound experts to assess the image quality achieved [23, 54, 56], only a few studies used structured testing protocols. Some studies assessed image quality according to a scoring system based on an Objective Structured Clinical Examination (OSCE), though they gave no information about when participants were deemed to have passed this test or the tools used were not described in a differentiated way [29, 47, 50]. Other studies used the Cardiac Ultrasound Structural Assessment Scale (CUSAS) score, others their own checklists, and still others measured the duration of the examination [23, 54, 56, 61]. However, all observed the result alone and provided no information about the processes of the examinations or difficulties encountered. To address these limitations, we adopted an assessment system based on DOPS, which enabled us to make a differentiated observation of sub-areas of competence [33, 39]. With this approach, we observed that one of the most common errors was poor image optimization. Furthermore, paramedics demonstrated greater proficiency in examining thoracic structures (lung, heart) compared to left-sided abdominal sections and the pelvis, consistent with findings from prior studies [22, 47, 51]. Our findings underscore the need for future training to prioritize certain sub-areas, such as thoracic sonography or image optimization.

A realistic self-assessment of skills is important for the safe use of POCUS. We therefore also recorded participants’ subjective views on their theoretical and practical skills. These increased significantly over the observation period, which is in line with other studies [56]. Our study additionally showed that the subjective skills before the start of the curriculum correlated strongly with the objective skills, which suggests subjects initially assessed themselves accurately. However, this correlation was no longer observed at the end of the curriculum. This finding could be well explained by the Dunning-Kruger effect [62]. It also highlights the importance of qualitative feedback at the end of a curriculum to promote a realistic self-assessment. These results also call for a long-term assessment of participants' retention of skills (e.g. after one year), which is a commonly used methodology in ultrasound education studies [63]. Follow-up results should be related to participants' clinical exposure and self-assessment.

Our study also used regression models to obtain more information about the influence of prior knowledge. The physician study group had performed a high number of ultrasound exams prior to their course, which would be expected in a clinical setting requiring regular encounters with sonographic scans and findings as part of study and work. This prior experience had an influence on the results of the theoretical and practical tests. Prior experience also affected the other two study groups, in which subjects exhibited less familiarity with POCUS and other forms of ultrasound. We suggest that integration of POCUS into paramedic training, and a regular use of POCUS in their work environment, can help future paramedics build up and maintain a basic understanding of the technique and thereby increase their general competence level even before specific training. Within the paramedic study group, prior attendance at a POCUS training course had a positive influence on results before the start of the curriculum. At the end of the curriculum, however, this influence was no longer measurable, which is an indication of the efficacy of the tailored curriculum.

### Discussion and acceptance of the developed curriculum

The high evaluation results for our curriculum indicate that the paramedic group embraced it willingly. This acceptance was also reflected in published studies that have evaluated POCUS curricula for paramedics [64, 65].

When medical curricula are being developed, appropriate content, duration, and a tailored design are essential factors for successful competence development [34]. Existing curricula for paramedics are extremely heterogeneous in terms of content and duration and are mostly based on training curricula for physicians [32]. Prior studies have concentrated on isolated aspects of POCUS (e.g. thoracic ultrasound, echocardiography, or the e-FAST protocol) for their course content, whereas we integrated several areas of application within our study. This enabled us to gain a differentiated view of the strengths and weaknesses of different POCUS aspects relevant to EMS. Furthermore, the duration of previously published curricula varied between two minutes and two months [32], with extremely brief curricula likely contributing to the failure to achieve primary endpoints in some studies [43, 44, 46, 59].

To compensate for paramedics’ lower level of prior knowledge and ultrasound experience, the duration of the curriculum in our study was extended in comparison with existing curricula [12]. Taking into account time efficiency, we incorporated a blended learning strategy [37]. This is employed with increasing frequency in ultrasound training and has already been used successfully to develop paramedics’ POCUS skills [30, 57, 65], but mostly utilizing Free Open Access Medical Education (FOAMed) content [23, 57]. The positive feedback about our blended learning approach, especially the webinar and the self-developed e-learning, highlights the future importance of this teaching strategy for paramedic ultrasound training. In this context, video-based training is a particularly good way of supporting the teaching of ultrasound skills [66, 67]. Furthermore, the use of blended learning meant that the duration of on-site sessions could be reduced to an acceptable minimum. This permitted greater focus on practical training, which is an important foundation for broad implementation in EMS [30, 57, 68, 69]. Apart from blended learning, the two remaining core didactic elements, “Case-based learning” and the “iPOCUS approach”, received positive evaluations from the participants and provided paramedics with a familiar learning atmosphere, which coincides with results from prior studies of other user groups [70].

The positive results of the evaluation and the associated acceptance of the curriculum indicate that future curricula are developed explicitly according to paramedics’ needs. This may enhance paramedics’ engagement with the learning and additionally result in standardization of the heterogeneous curricula [31, 32].

### Future prospects

The high motivation and positive attitude that paramedics exhibited towards POCUS as measured in our study indicate the need for further investigation of POCUS’ role and ideal implementation in paramedicine. Primarily, studies should consider how to implement POCUS in paramedic training. In addition, existing or newly created curricula should undergo certification procedures by professional associations for the purposes of quality assurance. Future curricula should focus on consolidating practical skills through clinical attachments in emergency or ultrasound departments, and should emphasise POCUS for prehospital use, preferably under qualified supervision and with the support of studies. Pre-existing or newly created institutions or committees of professional associations should address POCUS for paramedics directly, and, in dialogue with paramedics themselves, should consider possible strategies for best implementing POCUS in paramedicine.

### Limitations

This study did not include a control group without intervention, which is why randomization was not possible. Interpretation of the competence comparison is limited by the non-uniform curricula which the control groups had undergone. In addition, the practical competences between the groups were tested on a simulator, which increases comparability, but only allows limited conclusions to be drawn about the actual POCUS competence of the groups on humans. Furthermore, the practical application within the study group was only tested on healthy volunteers under training conditions, which also limits the translation to real patients. We developed the measuring instruments used (tests and DOPS) on the basis of recommendations and in consultation with experts, but they were not conclusively validated. Furthermore, we cannot exclude a relevant motivation bias within the study group in view of their voluntary participation in the curriculum and the study. A lack of long-term follow-up means we cannot make any assertion about the sustainability of the skills acquisition. The very small subgroups limit our analysis of influencing factors, which is why it is only possible to assume trends. Furthermore, we cannot exclude the possibility that other factors not recorded in the study had an influence on the results. Given that we did not implement a field trial involving the use of POCUS on patients, future studies are needed to evaluate the informative value of this study with regard to safety, practicability, and transferability to real-world use on patients.

## Conclusion

The results of this study deliver valuable evidence that, after completing a tailored curriculum, German paramedics can develop basic skills in POCUS that are comparable to those of other POCUS learners. These findings demand that more scientific attention be given to POCUS training for paramedics. The future implementation of ultrasound-specific teaching content in paramedic training offers opportunities to promote the inter-professional prehospital application of POCUS.

### Supplementary Information

Supplementary Material 1.

Supplementary Material 2.

Supplementary Material 3.

Supplementary Material 4.

Supplementary Material 5.

Supplementary Material 6.

Supplementary Material 7.

Supplementary Material 8.

Supplementary Material 9.

## Data Availability

Data cannot be shared publicly because of institutional and national data policy restrictions imposed by the Ethics committee since the data contain potentially identifying study participants’ information. Data are available upon request from the Johannes Gutenberg University Mainz Medical Center (contact via weimer@uni-mainz.de) for researchers who meet the criteria for access to confidential data (please provide the manuscript title with your enquiry).
