# Supplementary material for: Development and evaluation of a point-of-care ultrasound curriculum for paramedics in Germany – a prospective observational study and comparison
Source: BMC Med Educ. 2024 Jul 29;24:811. doi: 10.1186/s12909-024-05816-1 (PMC11285294; doi:10.1186/s12909-024-05816-1)
Supplement: Supplementary file 2 — Supplementary Material 2. [file 12909_2024_5816_MOESM2_ESM.pdf]

Supplement 2

a) Paramedics' time schedule of the on-site phase, duration of application

Time	Minutes	Topic
**On-site phase (Day 1)**		
08.30 – 08.45	15	Registration
08.45 – 09.00	15	Start and presentation of the program
09.00 – 09.45	45	Test[T2]
09.45 – 10.00	15	Lecture: Basics and iPOCUS
10.00 – 10.20	20	Lecture: Retroperitoneum (IVC and Aorta)
10.20 – 10.30	10	Coffee break
10.30 – 11.15	45	Hands-on training 1 – Rotation 1
11.15 – 12.00	45	Hands-on training 1 – Rotation 2
12.00 – 12.45	45	Hands-on training 1 – Rotation 3
12.45 – 13.00	15	Buffer
13.00 – 14.00	60	Lunch
14.00 – 14.20	20	Lecture: Body cavities
14.20 – 14.45	25	Lecture: Urinary tract
14.45 – 15.30	45	Hands-on training 2 – Rotation 1
15.30 – 16.15	45	Hands-on training 2 – Rotation 2
16.15 – 16.30	15	Coffee break
16.30 – 17.15	45	Hands-on training 2 – Rotation 3
17.15 – 18.00	45	Lecture: Ultrasound in cardiac arrest
18.00 – 18.30	30	Free practice
**On-site phase (Day 2)**		
08.30 – 08.45	15	Opening and program of the day
08.45 – 09.20	35	Lecture: Lung ultrasound
09.20 – 09.50	30	Lecture: RUSH protocol
09.50 – 10.00	10	Coffee break
10.00 – 10.40	40	Hands-on training 3 – Rotation 1
10.40 – 11.20	40	Hands-on training 3 – Rotation 2
11.20 – 12.00	40	Hands-on training 3 – Rotation 3
12.00 – 13.00	60	Lunch break
13.00 – 14.00	60	Hands-on training 4 – Rotation 1 (P-RUSH[T3])
14.00 – 15.00	60	Hands-on training 4 – Rotation 2 (P-SIM[T3])
15.00 – 16.00	60	Hands-on training 4 – Rotation 3 (Test[T3])
16.00 – 16.30	30	Evaluation[T3] and end of day 2

b) Personal practice time for each ultrasound application per participant:

- IVC: 11 minutes per participant
- Aorta: 11 minutes per participant
- FAST: 22 minutes per participant
- Lung (anterior and lateral views): 10 minutes per participant
- Heart (subxiphoid view): 10 minutes per participant
- Veins: 10 minutes per participant (+ extra time)
- Extra time during "free practice" per participant: 10 minutes per participant
- Practical assessments: 30 minutes per participant
