# Supplementary material for: Development and evaluation of a point-of-care ultrasound curriculum for paramedics in Germany – a prospective observational study and comparison
Source: BMC Med Educ. 2024 Jul 29;24:811. doi: 10.1186/s12909-024-05816-1 (PMC11285294; doi:10.1186/s12909-024-05816-1)
Supplement: Supplementary file 3 — Supplementary Material 3. [file 12909_2024_5816_MOESM3_ESM.pdf]

Supplement 3 - Excerpt of the theoretical test

Question 1

Label the numbers shown with the correct anatomical term.

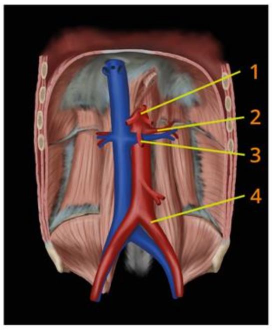

Answer
1:
2:
3:
4:

Question 2

Which ultrasound mode is shown on the left?

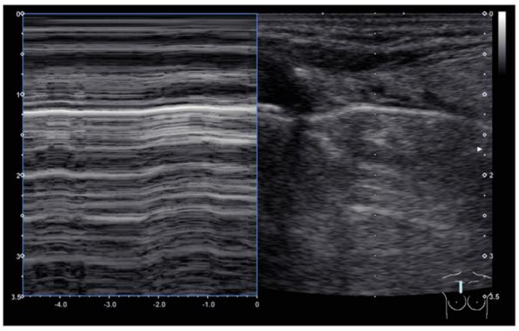

Answer

Question 3

Play the following ultrasound clip. Which aspects of image optimization should be performed in this clip?

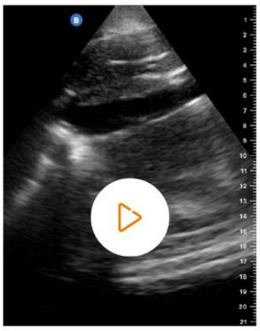

Answer

Question 4

Look at the transducer position shown in the picture and the figure next to it. Assign the correct anatomical direction names to numbers 1 to 4. Choose from the words below.

Answer
1:
2:
3:
4:
☐ patients right
☐ patients left
☐ cranial
☐ caudal
☐ ventral
☐ dorsal

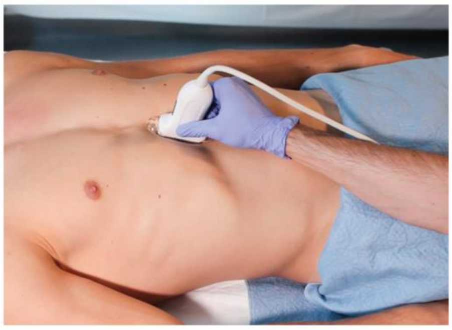

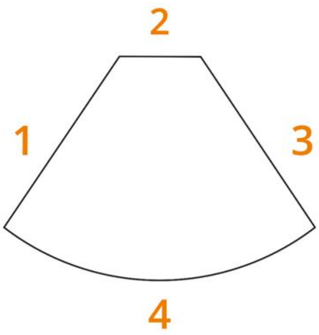

Question 5

Look at the transducer position shown on the picture and the ultrasound images next to it. Assign the appropriate ultrasound image to the displayed transducer position.

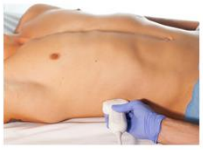

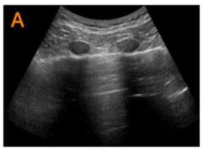

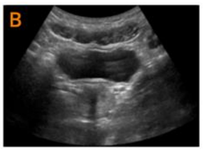

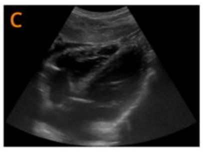

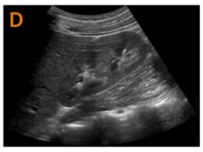

Single choice answer: ☐ A ☐ B ☐ C ☐ D

Question 6

Look at the transducer position shown on the picture and the ultrasound images next to it. Assign the appropriate ultrasound image to the displayed transducer position.

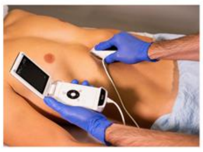

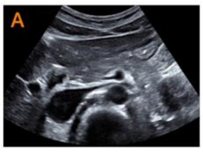

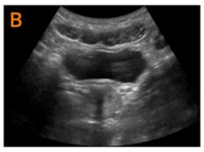

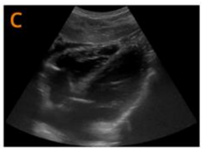

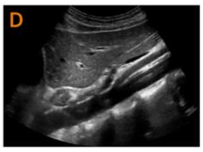

Single coice answer: ☐ A ☐ B ☐ C ☐ D

Question 7

Look at the ultrasound image and the transducer positions shown next it. Assign the correct transducer position to the displayed ultrasound image.

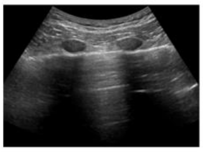

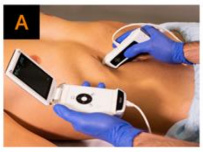

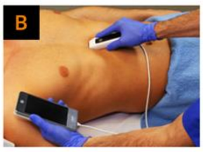

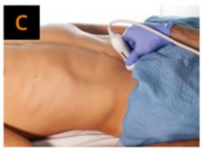

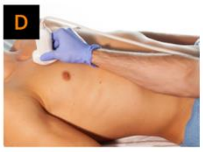

Single choice answer: ☐ A ☐ B ☐ C ☐ D

Question 8

Take a look at the following clip. It was acquired in the right mid axillary line, at the level of the epigastrium. Assign the correct term to the numbers displayed. If you see a pathologic finding, name it. If you do not see any pathological findings, enter a dash (-) under pathology.

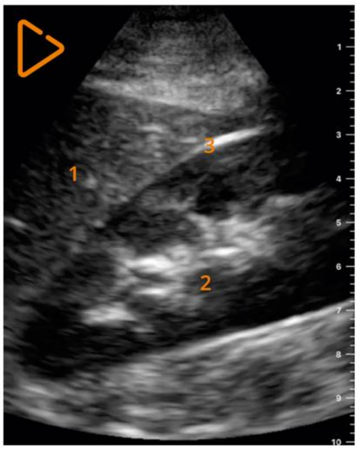

Answer
1:
2:
3:
Pathology:

Question 9

Take a look at the following clip. It was acquired in the right parasternal line, at the level of the 4th ICS. Assign the correct term to the numbers displayed. If you see a pathologic finding, name it. If you do not see any pathological findings, enter a dash (-) under pathology.

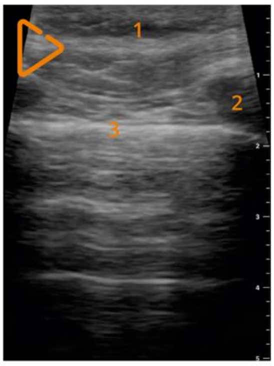

Answer
1:
2:
3:
Pathology:

Question 10

Take a look at the following clip. It was acquired in the median line cranial to the umbilicus. Assign the correct term to the numbers displayed. If you see a pathologic finding, name it. If you do not see any pathological findings, enter a dash (-) under pathology.

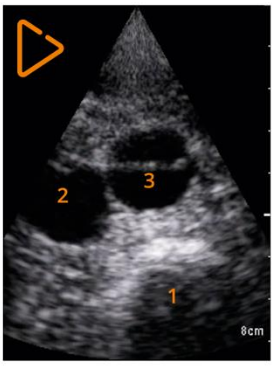

Answer
1:
2:
3:
Pathology:

Question 11

Take a look at the following clip. It was acquired in the cranial epigastrium. The transducer was tiled in direction of the chest. Assign the correct term to the numbers displayed. If you see a pathologic finding, name it. If you do not see any pathological findings, enter a dash (-) under pathology.

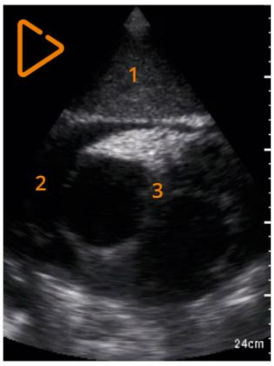

Answer
1:
2:
3:
Pathology:

Question 12

Take a look at the following clip. It was acquired in the right mid axillary line, at the level of the epigastrium. Assign the correct term to the numbers displayed. If you see a pathologic finding, name it. If
you do not see any pathological findings, enter a dash (-) under pathology.

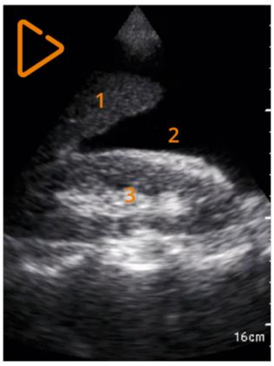

Answer
1:
2:
3:
Pathology:
