# Supplementary material for: Development and evaluation of a point-of-care ultrasound curriculum for paramedics in Germany – a prospective observational study and comparison
Source: BMC Med Educ. 2024 Jul 29;24:811. doi: 10.1186/s12909-024-05816-1 (PMC11285294; doi:10.1186/s12909-024-05816-1)
Supplement: Supplementary file 4 — Supplementary Material 4. [file 12909_2024_5816_MOESM4_ESM.pdf]

Supplement 4

a) Excerpt of the practical test P-Sim^{T3}

DOPS No. 1/3								
Heart							Pathology:	
Information text (Read out the following text slowly): „Your team resuscitates a 25-year-old patient in cardiac arrest and PEA. You decide to perform an ultrasound examination. Please examine the heart sonographically using the subxiphoid 4-chamber view and assess the findings."							Cardiac tamponade	
Performance assessment							Pathology recognition	
Procedure		Expectation	yes	verb	man		Item	Points
i	Indication	Names a specific question before the beginning	2 ☐	1 ☐	0 ☐		Recognizes pathology completely, can name or describe it independently.	3 ☐
P	Position	Places the transducer in the correct position	2 ☐	1 ☐	0 ☐			
O	Orientation	Checks the orientation of the transducer	2 ☐	1 ☐	0 ☐		Only partially recognizes the pathology or the extent of the pathology.	2 ☐
C	Correction	Corrects the position and optimizes the image correctly	2 ☐	1 ☐	0 ☐			
U	Ultrasound examination	Examines the target structure for the question	2 ☐	1 ☐	0 ☐		Recognizes the pathology only after verbal assistance. Help: "What does the area around the heart look like?"	1 ☐
S	Save and speak	Saves an image / clip and names the findings	2 ☐	1 ☐	0 ☐			
Performance		very good 7 - 6 - 5 - 4 - 3 - 2 - 1 insufficient					Does not recognize pathology / names pathology incorrect.	0 ☐
			Score	____ of 19				

Examiner instructions	
General instructions	10 minutes per DOPS. If unable to complete the task independently, please provide verbal (verb) and, if necessary, manual (man) assistance.
Rating	Green = Yes = The candidate performs the task completely correctly / optimally.<br>Yellow = verb = The candidate performed the task with difficulty, so that verbal help was necessary.<br>Red = man = the candidate was only able to perform the task with manual help from the examiner..
Objectives	At this station, participants are given the opportunity to perform a focused, heart-related ultrasound examination and interpret the findings. Both the examination procedure according to iPOCUS and the detection of findings are evaluated.
Notes	- It is not necessary to count the total points.<br>- The times must be strictly adhered to.<br>- Start by reading the information text and stop the time.

b) iPOCUS approach with specific details

iPOCUS approach (handout for participants)	
Indication	Check the indication strictly.<br>Define the question of your examination.<br>Set a time limit.
Position	Locate the anatomical landmark.<br>Apply sufficient ultrasound gel.<br>Position the transducer.
Orientation	Disconnect: left side of the image should darken!<br>Sagittal examination: disconnect cranially<br>Transverse examination: disconnect on the right
Correction	Visualize the target structure.<br>Adjust Depth, Gain and Focus.<br>Use inspiration / optimize patient position.
Ultrasound examination	Examine the target structure.<br>Evaluate across organ / structure borders.<br>Determine flow / perform measurements.
Save and speak	Save images and clips.<br>Inform the team.<br>Discuss the next steps.
