# Supplementary material for: Development and evaluation of a point-of-care ultrasound curriculum for paramedics in Germany – a prospective observational study and comparison
Source: BMC Med Educ. 2024 Jul 29;24:811. doi: 10.1186/s12909-024-05816-1 (PMC11285294; doi:10.1186/s12909-024-05816-1)

Supplement 5

a) Course structures of the different groups and time points of comparison assessments.

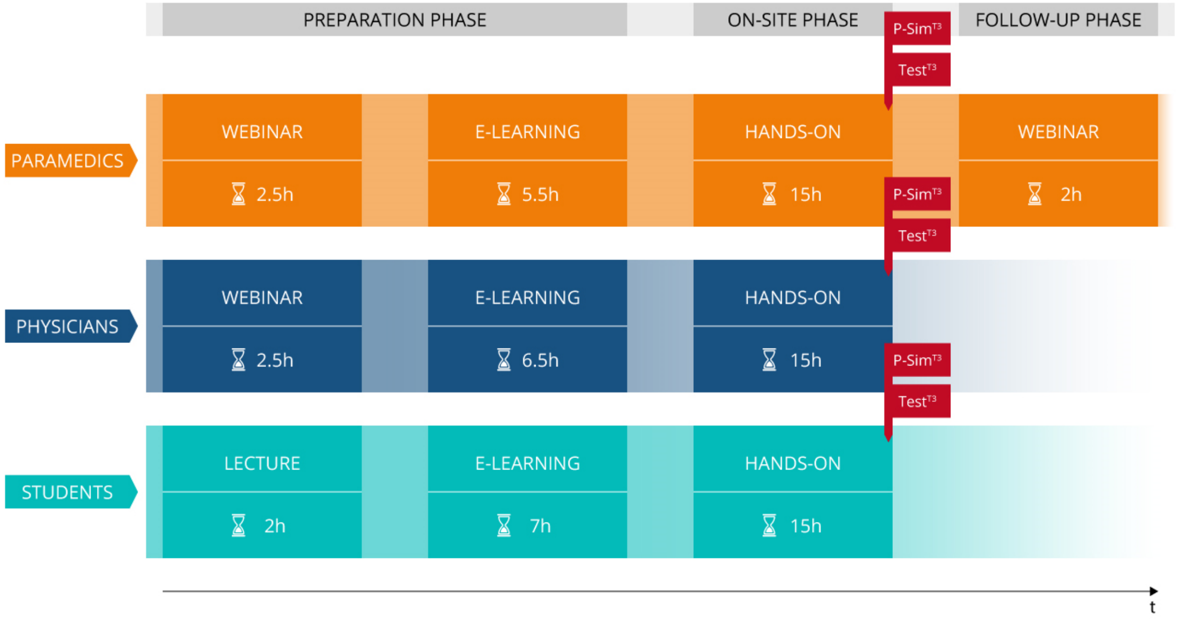

b) Illustration of participant in- and exclusion in the different groups.

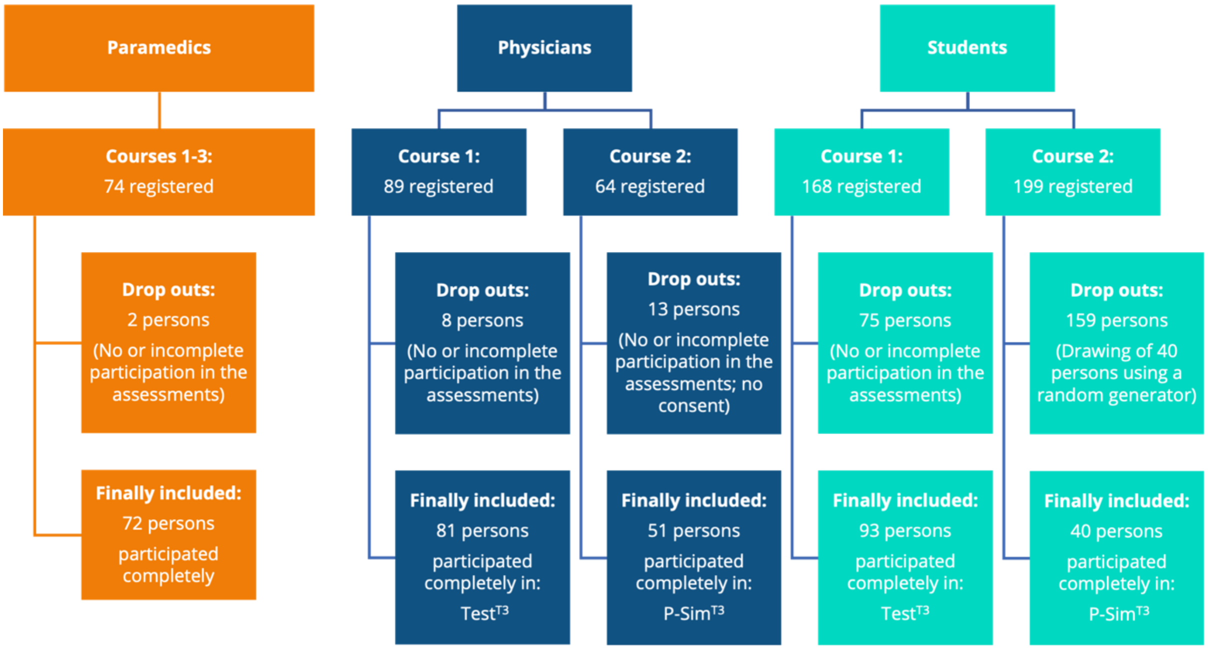

Supplement: Supplementary file 5 — Supplementary Material 5. [file 12909_2024_5816_MOESM5_ESM.pdf]
