# Supplementary material for: Development and evaluation of a point-of-care ultrasound curriculum for paramedics in Germany – a prospective observational study and comparison
Source: BMC Med Educ. 2024 Jul 29;24:811. doi: 10.1186/s12909-024-05816-1 (PMC11285294; doi:10.1186/s12909-024-05816-1)
Supplement: Supplementary file 6 — Supplementary Material 6. [file 12909_2024_5816_MOESM6_ESM.pdf]

Supplement 6 - Baseline of the study group and the comparison groups

Baseline items	Paramedics n=72	Physicians n=132	Students n=133
**Age** Mean (SD)			
	31.6	31.8	24.6
**Sex** n (%)			
Female	13 (18.1)	78 (59.1)	90 (67.7)
Male	59 (81.9)	54(40.9)	43 (32.3)
**Prior medical training**			
Yes	72 (100)	15 (11.4)	77 (57.9)
No	0 (0)	117 (88.6)	56 (42.1)
**Work experience** n (%)			
0 years	3 (4)	2 (1.5)	97 (72.9)
1 year	9 (12.5)	53 (40)	6 (4.5)
2 years	0	25 (18.9)	2 (1.5)
3 years	0	12 (9.1)	5 (3.8)
4 years	17 (23.6)	11 (8.3)	4 (3)
>=5 years	43 (59.7)	29 (22.0)	19 (14.3)
**Prior ultrasound course attendance** n (%)			
Yes	15 (20.8)	43 (32.6)	20 (15.0)
No	57 (79.2)	89 (67.4)	113 (85.0)
**Prior self-performed ultrasound studies** n (%)			
0	45 (62.5)	1 (0.8)	95 (71.4)
1-10	18 (25)	30 (22.7)	34 (25.6)
11-20	4 (5.6)	22 (16.7)	2 (1.5)
21-49	3 (4.2)	21 (15.9)	2 (15)
50-99	1 (1.4)	28 (21.2)	0 (0)
100-200	1 (1.4)	11 (8.3)	0 (0)
>200	0 (0)	19 (14.4)	0 (0)
**Physician position** n (%)			
Resident physician	-	111 (84.1)	-
Specialist physician	-	18 (13.6)	-
Attending physician	-	3 (2.3)	-
**Physician specialty** n (%)			
Internal Medicine	-	72 (54.5)	-
Surgery	-	22 (16.7)	-
General Medicine	-	17 (23.6)	-
Anaesthesiology	-	8 (11.1)	-
Other	-	13 (18.1)	-
**Prehospital Emergency Physician Qualification** n (%)			
Yes	-	9 (6.8)	-
No	-	123 (93.2)	-
**Students semester** n (%)			
First clinical (3rd year medical students)	-	-	126 (94.7)
Second clinical (3rd year medical students)	-	-	6 (4.5)
Third clinical (4th year medical students)	-	-	1 (0.8)
