# Supplementary material for: Development and evaluation of a point-of-care ultrasound curriculum for paramedics in Germany – a prospective observational study and comparison
Source: BMC Med Educ. 2024 Jul 29;24:811. doi: 10.1186/s12909-024-05816-1 (PMC11285294; doi:10.1186/s12909-024-05816-1)
Supplement: Supplementary file 7 — Supplementary Material 7. [file 12909_2024_5816_MOESM7_ESM.pdf]

Supplement 7 - Detailed baseline of the study group

Items	Evaluation[T1]
**Age** MW ± SD	
	31.6 ± 9.5
**Sex** n (%)	
Female	13 (18.1%)
Male	59 (81.9%)
**Highest emergency service qualification**	
Paramedic	65 (90.3%)
EMT-B	7 (9.7%)
**Federal state** n (%)	
Baden-Württemberg	20 (27.8%)
Hessen	13 (18.1%)
Nordrhein-Westfalen	13 (18.1%)
Berlin	6 (8.3%)
Rheinland-Pfalz	6 (8.3%)
Bayern	4 (5.6%)
Other	10 (13.9%)
**Work experience after certification** n (%)	
0 years	3 (4%)
1 year	9 (12.5%)
2 years	0
3 years	0
4 years	17 (23.6%)
≥5 years	43 (59.7%)
**Practical instructor** n (%)	
Yes	35 (48.6%)
No	37 (51.4%)
**Teacher at paramedic school** n (%)	
Yes	28 (38.9%)
No	44 (61.1%)
**Full-time prehospital employment** n (%)	
Yes	58 (80.6%)
No	14 (19.4%)
**Work on physician staffed response cars** n (%)	
Yes	44 (61.1%)
No	28 (38.9%)
**Ultrasound device available on ambulance** n (%)	
Yes	1 (1.4%)
No	71 (98.6%)
**Ultrasound device on physician staffed response car** n (%)	
Yes	40 (55.6%)
No	32 (44.4%)
**Prior ultrasound course attendance** n (%)	
Yes	15 (20.8%)
No	57 (79.2%)
Owner of a private ultrasound device n (%)	
Yes	10 (13.9%)
No	62 (86.1%)
**Total prior ultrasound examinations** n (%)	
0	45 (62.5)
1-10	18 (25)
11-20	4 (5.6)
21-49	3 (4.2)
50-99	1 (1.4)
100-200	1 (1.4)
