# Supplementary material for: Development and evaluation of a point-of-care ultrasound curriculum for paramedics in Germany – a prospective observational study and comparison
Source: BMC Med Educ. 2024 Jul 29;24:811. doi: 10.1186/s12909-024-05816-1 (PMC11285294; doi:10.1186/s12909-024-05816-1)
Supplement: Supplementary file 8 — Supplementary Material 8. [file 12909_2024_5816_MOESM8_ESM.pdf]

Supplement 8 - Motivation, self-assessment and learning objectives (MV + SD in %), Likert Scale 1-7 (presented after transformation into percentages).

Item	Evaluation[T1]	Evaluation[T3]	p-value
**Motivation and attitude (total score)**	86.7 (8.0)	91.1 (7.7)	<0.001
Interest in prehospital ultrasound	97.2 (7.8)	98.0 (6.5)	0.5184
Usefulness of prehospital ultrasound	88.9 (14.5)	93.1 (14.0)	0.07973
Questions can be answered by prehospital ultrasound	91.9 (12.9)	94.9 (11.1)	0.1421
Equip ambulances with ultrasound devices as quickly as possible	75.3 (22.6)	84.3 (21.4)	0.01575
Paramedics should learn and use ultrasound	91.1 (12.2)	94.4 (14.1)	0.1266
Improvement of prehospital patient care through ultrasound	91.7 (13.1)	93.4 (12.1)	0.4096
Implementation of ultrasound in Paramedic training	82.5 (22.4)	88.1 (21.5)	0.1331
Training and use of ultrasound by emergency physicians	97.6 (10.2)	98.0 (10.8)	0.8245
Use of prehospital ultrasound in trauma patients could be crucial	80.6 (21.2)	85.1 (17.7)	0.1656
**Self-assessment (total score)**	41.9 (16.5)	78.4 (8.1)	<0.001
**Total score of theoretical skills**	**47.3 (16.9)**	**77.9 (13.2)**	**<0.001**
Anatomy knowledge	63.2 (19.5)	78.1 (17.6)	<0.001
Basic physical ultrasound principles	52.8 (21.5)	76.6 (16.6)	<0.001
Image generation	54.4 (19.7)	81.1 (16.7)	<0.001
Orientation	49.8 (22.1)	80.4 (15.8)	<0.001
Artifacts	35.3 (21.3)	76.8 (16.3)	<0.001
Knowledge of standard sections	36.9 (22.8)	81.6 (17.7)	<0.001
Recognition of pathologies	43.9 (23.9)	72.4 (16.5)	<0.001
Sonoanatomical mapping	43.3 (23.0)	78.8 (14.2)	<0.001
**Total Score of practical skills**	**37.1 (19.5)**	**78.2 (12.6)**	**<0.001**
Transducer handling	43.5 (22.9)	76.1 (15.7)	<0.001
Image optimization	40.8 (24.5)	78.6 (17.0)	<0.001
Patient guidance	45.8 (24.6)	75.2 (17.1)	<0.001
Generate standard sections	36.4 (22.0)	77.9 (14.4)	<0.001
Structure examination	46.7 (24.4)	75.6 (14.9)	<0.001
Examination of the IVC	29.8 (21.0)	83.9 (16.2)	<0.001
Examination of the abdominal aorta	31.2 (23.9)	78.0 (16.9)	<0.001
Performance of the FAST protocol	33.9 (23.1)	77.5 (15.0)	<0.001
Examination of the lungs	35.3 (22.5)	82.5 (16.8)	<0.001
Examination of the heart	36.4 (21.8)	73.7 (16.9)	<0.001
**Learning objectives**	91.2 (7.7)	87.7 (8.1)	-
Improvement of anatomical knowledge	82.3 (17.9)	86.7 (20.2)	-
Development of a basic understanding of sonography	95.3 (13.5)	97.0 (11.3)	-
Readiness for prehospital use	86.9 (16.6)	76.7 (16.8)	-
Safety in the use of ultrasound on emergency patients	88.9 (16.0)	76.9 (16.5)	-
Interpretation of pathological findings	92.5 (13.2)	78.1 (19.2)	-
Learn about possible applications for prehospital use	94.3 (10.9)	89.3 (14.2)	-
Exchange with other ultrasound enthusiasts	88.7 (17.4)	90.7 (16.4)	-
Expansion of the personal competence horizon	98.8 (4.3)	98.6 (6.4)	-
