# Supplementary material for: Development and evaluation of a point-of-care ultrasound curriculum for paramedics in Germany – a prospective observational study and comparison
Source: BMC Med Educ. 2024 Jul 29;24:811. doi: 10.1186/s12909-024-05816-1 (PMC11285294; doi:10.1186/s12909-024-05816-1)
Supplement: Supplementary file 9 — Supplementary Material 9. [file 12909_2024_5816_MOESM9_ESM.pdf]

Supplement 9 - Comparison of practical and theoretical competence between the groups

a) Practical competence comparison			
**Item**	**Paramedics**	**Physicians**	**Students**
**Total score** Mean (SD)	83.8 (6.6)	88.3 (6.1)	84.1 (6.2)
compared to Paramedics		p<0.001	p=0.86
compared to Physicians	p<0.001		p<0.01
compared to Students	p=0.86	p<0.01	
**Position** Mean (SD)	96.7 (7.8)	96.0 (8.0)	91.5 (8.6)
compared to Paramedics		p=0.63	p<0.01
compared to Physicians	p=0.63		p<0.01
compared to Students	p<0.01	p<0.01	
**Orientation** Mean (SD)	94.9 (9.2)	94.1 (9.9)	92.5 (14.6)
compared to Paramedics		p=0.70	p=0.27
compared to Physicians	p=0.70		p=0.49
compared to Students	p=0.27	p=0.49	
**Correction** Mean (SD)	72.0 (17.1)	77.1 (14.0)	80.0 (16.4)
compared to Paramedics		p=0.08	p=0.01
compared to Physicians	p=0.08		p=0.40
compared to Students	p=0.01	p=0.40	
**Ultrasound exam** Mean (SD)	89.0 (13.2)	93.1 (11.7)	90.4 (11.9)
compared to Paramedics		p=0.08	p=0.59
compared to Physicians	p=0.08		p=0.30
compared to Students	p=0.59	p=0.30	
**Save and speak** Mean (SD)	78.4 (18.6)	76.5 (15.2)	73.8 (18.4)
compared to Paramedics		p=0.60	p=0.18
compared to Physicians	p=0.60		p=0.46
compared to Students	p=0.18	p=0.46	
**General impression** Mean (SD)	78.1 (9.2)	85.6 (8.2)	81.3 (7.9)
compared to Paramedics		p<0.001	p=0.06
compared to Physicians	p<0.001		p=0.02
compared to Students	p=0.06	p=0.02	
**Pathology recognition** Mean (SD)	88.7 (12.0)	95.9 (7.3)	83.8 (11.4)
compared to Paramedics		p<0.001	p=0.02
compared to Physicians	p<0.001		p<0.001
compared to Students	p=0.02	p<0.001	

b) Theoretical competence comparison			
**Item**	**Paramedics**	**Physicians**	**Students**
**Total score incl. Pathologies**	82.9 (9.2)	81.0 (8.7)	76.7 (8.5)
compared to Paramedics		p=0.18	p<0.001
compared to Physicians	p=0.18		p<0.01
compared to Students	p<0.001	p<0.01	
**Anatomy**	91.1 (10.8)	87.7 (10.2)	94.2 (9.1)
compared to Paramedics		p=0.036	p=0.051
compared to Physicians	p=0.036		p<0.001
compared to Students	p=0.051	p<0.001	
**Basics**	89.2 (14.0)	84.0 (14.1)	86.9 (10.6)
compared to Paramedics		p=0.01	p=0.24
compared to Physicians	p=0.01		p=0.15
compared to Students	p=0.24	p=0.15	
**Orientation**	95.6 (10.7)	95.1 (9.8)	94.6 (10.3)
compared to Paramedics		p=0.77	p=0.56
compared to Physicians	p=0.77		p=0.78
compared to Students	p=0.56	p=0.78	
**Structure recognition**	83.6 (12.6)	83.9 (12.1)	81.4 (13.3)
compared to Paramedics		p=0.88	p=0.27
compared to Physicians	p=0.88		p=0.20
compared to Students	p=0.27	p=0.20	
**Pathologies**	74.1 (12.1)	72.4 (12.2)	59.3 (12.2)
compared to Paramedics		p=0.39	p<0.001
compared to Physicians	p=0.39		p<0.001
compared to Students	p<0.001	p<0.001	
